# Epidemiological, Clinical, and Laboratory Features of Children with SARS-CoV-2 in Ukraine

**DOI:** 10.34763/jmotherandchild.20232701.d-23-00012

**Published:** 2023-08-07

**Authors:** Tetiana Harashchenko, Tetiana Umanets, Volodymyr Podolskiy, Tetiana Kaminska, Yuriy Marushko, Vasily Podolskiy, Volodymyr Lapshyn, Yurii Antypkin

**Affiliations:** Department of Respiratory Diseases and Allergy in Children, SI “Institute of Pediatrics, Obstetrics and Gynecology named after Academician O.M. Lukyanova, National Academy of Medical Sciences of Ukraine”, Kyiv, Ukraine; Department of Pediatrics, CNE “Kyiv City Children's Clinical Infectious Disease Hospital”, Kyiv, Ukraine; Department of Pediatrics, National Medical University named after O.O. Bogomolets, Kyiv, Ukraine

**Keywords:** child, clinical features, COVID-19, infection, SARS-CoV-2

## Abstract

**Introduction:**

In December 2019, the Chinese city of Wuhan reported the first cases of pneumonia from a new type of beta coronavirus named SARS-CoV-2. In the early days of the COVID-19 outbreak, paediatric patients were thought to be immune to the new virus; however, further studies have shown people of all ages to be susceptible to the virus.

**Objective:**

Identify and describe the clinical and epidemiological features of COVID-19 among hospitalized children in Ukraine.

**Materials and Methods:**

Retrospective study of 171 children aged 2 months to 18 years who were hospitalized with laboratory-confirmed SARS-CoV-2.

**Results:**

Most patients in the study had a moderate progression of the disease (77.78%, or n=133), whereas a severe course was noted in 22.22% (n=38). Across age groups, children aged 6–12 was the predominant age group affected (35.67%, or n=61). The most common symptoms were fever in 88.2% of patients, sore throat in 69.2% and cough in 60.9%. Symptoms associated with dyspnoea and cyanosis were significantly more common in children with the severe course (p<0.05). Almost half of children had at least one comorbidity, the most prevalent being chronic tonsillitis (11.8% of patients) and anemia (6.5% of patients). A positive correlation (r=0.7 p<0.05) was found between CRP levels and COVID-19 severity. X-ray changes in the lungs were present in 76.61% of examined children and ground-glass opacity symptom was registered in 50.88%.

**Conclusions:**

COVID-19 among hospitalized children in Ukraine usually has a moderate course of illness and a good prognosis.

## Introduction

In December 2019, the Chinese city of Wuhan reported the first cases of pneumonia of unknown cause. The causative agent was later found to be a new type of beta coronavirus named severe acute respiratory syndrome coronavirus 2 (SARS-CoV-2). On February 11, 2020, the World Health Organization (WHO) officially named the disease Coronavirus disease 2019 (COVID-19) [2]. Since the first recorded case, COVID-19 has rapidly spread around the world and on March 11, 2020, WHO announced that COVID-19 had reached pandemic status [3]. The first case of COVID-19 in Ukraine was recorded on March 3, 2020, and the number of cases has gradually increased, just like in other parts of the world, among people of all ages.

In the early days of the COVID-19 outbreak, paediatric patients were considered immune to the new virus, but further studies have shown that people of all ages are susceptible to SARS-CoV-2 infection. Currently, children under 18 years of age account for 8.5% of total SARS infections [3,4]. The course of COVID-19 in children is relatively milder and has a better prognosis compared to adults. Almost 50% of children with COVID-19 are either asymptomatic or experience mild symptoms. However, some children require hospitalization and/or oxygen support, and some may develop multisystem inflammatory syndrome (MIS-C) [5,6]. According to preliminary studies, the clinical course in children varies from asymptomatic or mild to severe pneumonia with respiratory failure. SARS-CoV-2 has an incubation period of 1–14 days. In most patients, symptoms appear between the third and seventh day after the exposure [3]. Clinical manifestations are usually non-specific, the most common being fever, dry cough, myalgia, headache, abdominal pain, and diarrhoea [7].

Currently, as the review of the available international indexed publications demonstrates, there are no published studies specifically on COVID-19 progression among children in Ukraine.

### Objectives

Describe the demographic, epidemiological, clinical, radiological and laboratory features of COVID-19 progression in hospitalized children in Ukraine.

## Material and methods

Retrospective study of 171 randomly selected medical histories of patients aged 2 months to 18 years who were hospitalized with COVID-19 at Kyiv City Children's Clinical Hospital of Infectious Diseases Number 12 from July to November 2020.

The data for the research were collected from the paper-based patient medical histories. All the data obtained were entered in an electronic database for further calculation, in particular: patient demographics (age, sex); date of exposure; method of infection; clinical characteristics – severity of symptoms; comorbidities; laboratory test results such as leukocytes, lymphocytes, eosinophils, hemoglobin, erythrocyte sedimentation rate (ESR), C-reactive protein (CRP), alanine aminotransferase (ALT), aspartate aminotransferase (AST), urea, creatinine; and results of chest radiography and lung imaging description.

On admission, all children had a blood panel done to determine the number of erythrocytes, leukocytes, lymphocytes, eosinophils, and ESR and hemoglobin level; a blood chemistry test was done to measure CRP, ALT and AST in 165 children; creatinine and urea measurements were performed in 137 children. A chest X-ray was performed in all children and was the only diagnostic imaging modality used in these patients.

The diagnosis was established based on the detection of viral particles by real-time nasopharyngeal and oropharyngeal reverse transcription polymerase chain reaction (RT-PCR) which is the gold standard in coronavirus diagnostics per the WHO guidelines [8]. The hospital laboratory, which is certified by Ukraine's Ministry of Health. Performed a SARS-CoV-2 nucleic acid test in all patients. The diagnosis date was the date of receipt of a positive test result. Fever was defined as body temperature ≥38°C according to the Centers for Disease Control and Prevention guidelines.

Continuous variables were presented as the mean with standard deviation, whereas categorical variables were expressed as a number and a percentage. The chi-square test and Student's t-test were used to assess statistical significance in variables. Authenticity was established according to the generally accepted practice in healthcare – 95%, p < 0.05. All data were entered in Microsoft Excel to perform calculations, processing, and analysis.

### *Ethics committee approval*.

Ethical approval for the study was obtained from the bioethics committee of the State Institution “Institute of Paediatrics, Obstetrics and Gynecology named after Academician O.M. Lukyanova, National Academy of Medical Sciences of Ukraine”, protocol no. 28 (15.10.2020). Informed consent was obtained from patients.

## Results

Demographic and epidemiological features of pediatric patients are shown in Table 1. Among 171 hospitalized children, boys accounted for 50.3% (n=86), and girls for 49.7% (n=85). According to the WHO classification [9], children were divided into two groups depending on the COVID-19 severity: 1 – children with moderate illness (77.78%, or n=133); and 2 – children with severe illness (22.22%, or n=38). In the second group, five children were in the intensive care unit and one child needed oxygen support. Fatal cases were not recorded. There was no statistical difference in gender between the groups (p=0.379).

**Table 1.** Demographic Characteristics of Pediatric Patients

		**Moderate (*n*=133)**		**Severe (*n*=38)**		**Total cases (*n*=171)**	
		
		***n%***	***%***	***n***	***%***	***n***	***%***
Sex	Boys	64	48.12	22	57.89	86	50.3
	Girls	69	51.88	16	42.11	85	49.7
Age distribution, years	Infants (2 months – 1 year)	13	9.77	7	18.42	20	11.69
	Toddlers (1 – 3 years)	10	7.52	15	39.47	25	14.62
	Preschool (3 – 5 years)	6	4.51	5	13.16	11	6.43
	Middle Childhood (6 – 12 years)	57	42.86	4	10.53	61	35.67
	Adolescence (13 – 18 years)	47	35.34	7	18.42	54	31.58

Children were stratified by age according to widely accepted developmental stages: infants (2 months – 1 year) – 11.69% of patients (n=20); toddlers (1–3 years) – 14.62% (n=25); preschool (3–5 years) – 6.43% (n=11); middle childhood (6–12 years) – 35.67% (n=61); adolescence (13–18 years) – 31.58% (n =54) [10]. The mean age of the cohort study patients was 8.41 years (range 0.2 – 18), with children aged 6 to 12 years-old being the predominant age group (35.67%, or n=61).

In the patient cohort, the age group of 6–18-year-olds was identified as the prevailing group. School-age children diagnosed with COVID-19 were more likely to require hospitalization compared to preschool children and infants. The severe illness was significantly more often recorded in children from ages 1 to 3 (p<0.0001).

Among 171 children, we were able to identify that the primary source of infection with SARS-Cov-2 in 70.17% of children (n=120) was close contact with a patient with COVID-19. 101 children (59.06%) were infected with SARS-Cov-2 at home through exposure to family members who live in the same household (parents, sisters/brothers), and 19 children (11.11%) were infected outside the family at school or extracurricular activities. It is statistically significant that children were more likely to become infected at home (p<0.05). In 29.83% of patients (n=51) it was not possible to establish the source of infection.

Children with COVID-19 were reported to have a wide range of clinical manifestations: fever, sore throat, cough, rhinitis, myalgia, lymphadenopathy, wheezing, headaches, diarrhoea, anosmia, ageusia, dyspnoea, and cyanosis (Table 2). Indications for hospitalization were severe intoxication syndrome, fever, and dehydration. We analyzed the clinical manifestations of COVID-19 in hospitalized children and established that fever was the most common symptom among hospitalized children in Ukraine. Febrile temperature was recorded in 87.13% of children (n=149), with its average being 38.4°C (38.0°C–40°C). Sore throat was reported in 69.59% of patients (n=119); cough in 61.40% (n=105); and rhinitis in 58.48% (n=100). Less than half of the children had such symptoms as lymphadenopathy – 30.99% (n=53); wheezing – 29.82% (n=51); and dermatological changes, in particular, urticaria, maculopapular rash or vesicular rash, and erythema multiforme – 9.94% (n=17). Myalgia occurred in 39.77% of children (n=68), whereas headaches were observed in 22.81% of cases (n=39) and more often in children with the severe disease progression (p<0.005), which indicates a more pronounced intoxication syndrome. Diarrhoea was more common in children with severe illness (21.63%, or n=37) compared to the moderate group (15.79%, or n=21) (p=0.00001). Positive pulmonary symptoms (dyspnoea, cyanosis) were more common in children with severe course of the disease (p<0.005). Cyanosis was detected in five cases of COVID-19 among hospitalized children, all of whom had progressed to severe illness. Shortness of breath was detected in 14 patients, of whom 12 had the severe course of the disease. Symptoms such as anosmia and ageusia, which are among the most common COVID-19 complaints in adults, are less common in children. In our study, anosmia was reported in 10.53% of patients (n=18) and ageusia in 5.85% (n=10). In children with severe COVID-19, the following symptoms were most frequently manifested: fever in 92.11% of patients; cough in 73.68%; and rhinitis in 71.05%. None of the children with the severe course had dermatological changes, and only one child was reported to have ageusia and anosmia.

**Table 2.** Clinical Features of Children

**Symptoms**	**Symptom presence**	**Moderate (*n*=133)**		**Severe (*n*=38)**		**Total (*n*=171)**		**χ^2^ test**	***p*-value**
		
		***n***	**%**	***n***	**%**	***n***	**%**		
Fever	<38°c	114	85.71	35	92.11	149	87.13	0.58	0.44
		19	14.29	3	7.89	22	12.87		
Sore throat	+	90	67.67	29	76.32	119	69.59	0.67	0.41
	−	43	32.33	9	23.68	52	30.41		
Cough	+	77	57.89	28	73.68	105	61.40	2.47	0.11
	−	56	42.11	10	26.32	66	38.60		
Rhinitis	+	73	54.89	27	71.05	100	58.48	2.54	0.11
	−	60	45.11	11	28.95	71	41.52		
Myalgia	+	42	31.58	26	68.42	68	39.77	15.24	0.0001^*^
	−	91	68.42	12	31.58	103	60.23		
Lymphadenopathy	+	37	27.82	16	42.11	53	30.99	2.19	0.13
	−	96	72.18	22	57.89	118	69.01		
Wheezing	+	41	30.83	10	26.32	51	29.82	0.11	0.73
	−	92	69.17	28	73.68	120	70.18		
Headache	+	24	18.05	15	39.47	39	22.81	6.5	0.01^*^
	−	109	81.95	23	60.53	132	77.19		
Diarrhea	+	21	15.79	16	42.10	37	21.63	10.56	0.001^*^
	−	112	84.82	22	57.90	134	78.36		
Anosmia	+	17	12.78	1	2.63	18	10.53	2.24	0.13
	−	116	87.22	37	97.37	153	89.47		
Dermatological changes	+	17	12.78	0	0.00	17	9.94	2.24	0.13
	−	116	87.22	38	100.00	154	90.06		
Dyspnea	+	2	1.50	12	31.58	14	8.19	31.67	0.00001^*^
	−	131	98.50	26	68.42	157	91.81		
Ageusia	+	9	6.77	1	2.63	10	5.85	0.32	0.57
	−	124	93.23	37	97.37	161	94.15		
Cyanosis	+	0	0.00	5	13.16	5	2.92	10.02	0.001^*^
	−	133	100.00	33	86.84	166	97.08		

* *significant.*

Of the total number of children, more than half, or 51.46% (n=88), had at least one comorbidity. 52.04% of patients (n=89) had a history of recurrent respiratory infections (more than six episodes of respiratory infections per year) [11]. The most common comorbidities were chronic tonsillitis – identified in 20 children (11.7%); CMV carriers – 11 (6.43%); anaemia – 11 (6.43%); and atopic dermatitis – 9 (5.23%) (Table 3). 65.8% of children (n=25) with severe COVID-19 had at least one comorbidity. No statistical significance was found between the COVID-19 severity in children with and without comorbidities (p=0.11).

**Table 3.** Comorbidities

**Comorbidities**	**Moderate course (*n*=133)**		**Severe course (*n*=38)**		**Total (*n*=171)**		***p*-value**
		
	***n***	**%**	***n***	**%**	***n***	**%**	
RRI	60	45.11	29	76.31	89	52.04	0.7
Chronic tonsillitis	15	11.28	5	13.16	20	11.7	0.7
CMV	5	3.76	6	15.79	11	6.43	0.9
Anemia	6	4.51	5	13.16	11	6.43	0.5
Atopic dermatitis	7	5.26	4	10.52	9	5.23	0.6
Neurological disorder	3	2.25	5	13.16	8	4.68	0.5
Urinary tract diseases	6	4.51	2	5.26	8	4.68	0.6
Allergy	6	4.51	1	2.63	7	4.09	0.9
Congenital heart disease	5	3.76	1	2.63	6	3.51	0.9
Gastrointestinal diseases	3	2.25	2	5.26	5	2.92	0.5
Obesity	3	2.25	2	5.26	5	2.92	0.5
Epilepsy	2	1.5	3	7.89	5	2.92	0.8
Asthma	3	2.25	1	2.63	4	2.34	0.9
Congenital bone disorders	0	0	2	5.26	2	1.17	0.3
Oncological disease	2	1.5	0	0	2	1.17	0.3
Diabetes	1	0.75	0	0	1	0.58	0.3
HIV	1	0.75	0	0	1	0.58	0.3
UCTD	1	0.75	0	0	1	0.58	0.3

*RRI - recurrent respiratory infection, CMV – Cytomegalovirus, HIV - human immunodeficiency virus, UCTD - Undifferentiated Connective Tissue Dysplasia*

The laboratory test results are presented in Table 4. Children with the severe course were significantly more likely to develop erythropenia (p=0.001) compared to children with moderate disease. No severe course was recorded in children with elevated hemoglobin levels (c=0.001). Leukocytosis was determined in 18.71% of children; leukopenia in 22.22%; lymphocytosis in 7.02%; lymphopenia in 68.42%; an accelerated erythrocyte sedimentation rate in 9.36%; and an increased level of eosinophils in 1.75%. However, no statistical relationship between these indicators and disease severity was established (p>0.05). Elevated CRP was observed in 91 hospitalized children (53.21%), significantly more often in the severe group (p=0.001). In the group of children with moderate severity, 81.53% of children had an increased AST level, against 48.57% of children with the severe COVID-19 progression. It was possible to establish a statistical correlation between the level of AST (p=0.001) and the COVID-19 severity. No significant statistical difference was recorded across other biochemical parameters, such as ALT, creatinine, and urea.

**Table 4.** Laboratory Test Changes in Pediatric Patients with COVID-19

**Parameter**	**Reference values**	**Moderate (*n*=133)**		**Severe (*n* =38)**		**Total (*n*=171)**		***p*-value**
		
		***n***	**%**	***n***	**%**	***n***	**%**	
RBC (*10^12^ cells/L)	<3.5	2	1.50	3	7.90	5	2.92	0.001^*^
	3.5–5.0	71	53.38	22	57.90	93	54.38	0.6
	>5.0	60	45.11	13	34.21	73	42.70	0.2
HGB *(g/L)*	<110	6	4.51	1	2.63	7	4.09	0.5
	110–140	74	55.64	37	97.37	111	64.91	0.001^*^
	>140	53	39.85	0	0.00	53	30.99	0.001
WBC (*10^9^ cells/L)	<5.5	29	21.80	9	23.68	38	22.22	0.6
	5.5–12	81	60.90	20	52.63	101	59.06	0.3
	>12	23	17.29	9	23.68	32	18.71	0.1
Lymphocytes *(%)*	<45	93	69.92	24	63.16	117	68.42	0.4
	45–65	32	24.06	10	26.32	42	24.56	0.7
	>65	8	6.02	4	10.53	12	7.02	0.4
Eosinophils *(%)*	<0.5	2	1.50	1	2.63	3	1.75	0.4
	0.5–7	128	96.24	37	97.37	165	96.49	0.6
	>7	3	2.26	0	0.00	3	1.75	0.1
ERS *(mm/h)*	<10	119	89.47	36	94.74	155	90.64	0.2
	>10	14	10.53	2	5.26	16	9.36	0.2
CRP *(mg/L)*	<3	72	54.14	8	21.05	80	46.78	0.001^*^
	>3	61	45.86	30	78.95	91	53.22	
ALT *(IU/L)*	<40	116	89.23	32	91.48	148	86.59	0.6
	>40	14	10.76	3	8.57	17	10.30	0.6
AST *(IU/L)*	<40	106	81.53	17	48.57	123	75.54	0.001^*^
	>40	24	18.46	18	51.42	42	25.45	
Creatinine *(mmol/L)*	<50	65	60.74	25	83.33	91	66.42	0.01
	>50	42	39.25	5	16.66	46	35.57	
Urea *(mmol/L)*	2.5–6.5	95	88.78	25	83.33	120	87.59	0.5
	>6.5	12	11.21	5	16.66	17	12.40	0.5

* *significant.*

*RBC – red blood cells, HGB – Hemoglobin, WBC – White blood cells, ERS - Erythrocyte Sedimentation Rate, CRP – C-reactive protein, ALT - alanine aminotransferase, AST - aspartate aminotransferase.*

A chest X-ray examination was performed in all children. This was the only diagnostic imaging modality used in these patients. The most frequent changes observed in X-ray examinations were as follows: unilateral pneumonia; bilateral pneumonia; ground-glass opacity; and bronchitis. About half of the children had ground-glass opacity symptom: 60.16% (n=24) in the severe group, and 47.37% (n=63) in the moderate group. Unilateral pneumonia was observed with equal frequency in both groups, in about 12.28% of cases. Bilateral pneumonia was visualized in 7.60% of cases, but significantly more often in the severe group: 21.05% versus 3.73%. X-ray signs of bronchitis were reported only in children with moderate illness – 10 patients (7.52%).

In 23.39% of children (n=40), no changes were recorded according to chest X-ray examination. Among paediatric patients with severe disease, radiological changes were registered more frequently and observed in 37 out of 38 patients, compared to children with moderate COVID-19 progression (p=0.001).

## Discussion

Our study of the epidemiological, clinical, and laboratory features of the progression of COVID-19 in hospitalized children in Ukraine contributes to a deeper understanding of the age-specific peculiarities of the disease among children in Ukraine. The survey of 171 children showed that in the patient cohort, children with moderate severity prevailed – 77.78%. Similar results were described by researchers from other countries [12]. The patient distribution by gender revealed an approximately equal number of boys and girls – 50.3% and 49.7%, respectively. 64 boys manifested moderate COVID-19 severity against 69 girls, while severe course of the disease was observed in 22 boys against 16 girls; yet there was no statistically significant gender difference in children with moderate and severe progression (p> 0.05).

In our study, schoolchildren of ages 6 to 18 were the predominant group across the age groups, representing 67.25% of all examined patients. Ding et al. reported similar results [13]. Such results may be associated with less epidemiological control in primary and secondary schools compared to preschools, as well as prolonged exposure of children to poorly ventilated rooms. It is also worth noting that younger children may not have been included in our study because they had a milder course of the disease and did not require hospitalization.

Epidemiological contact was determined in 88.89% of the examined patients. It is statistically significant that children were more likely to be infected with SARS-CoV-2 virus at home than in the community (p<0.05). Ding et al. [13] and Hoang et al. [14] reported similar data in their respective studies.

In our study, the most common symptoms were fever (88.2% of cases), sore throat (69.2%) and cough (60.9%). Also, other researchers in their studies described same the most common symptoms (Hoang et al.; Xu et al.) [14, 15]. Additionally, available sources describe that common symptoms in more than 24% of children with COVID-19 are myalgia and headache [14, 16]. In our study cohort, myalgia was reported in 39.77% of cases (n=68), whereas headache was observed in 22.81% of children (n=39), most frequently in those with a severe course (p<0.05). Other studies describe these symptoms as markers of systemic inflammation and cytokine response [17]. Anosmia and ageusia, often observed in adults suffering from COVID-19, are much less common in paediatric patients. This may be explained by the inability of young children to describe and report these symptoms to the doctor, as well as the lack of valid diagnostic tests to evaluate anosmia and ageusia in pediatric patients [18]. Among the paediatric patients in our study, we identified anosmia in 10% of cases and ageusia only in 5.91%; both were more likely in children with a moderate, rather than severe, course. A number of preliminary studies describe that Olfactory and Gustatory Dysfunction is a marker of good prognosis of COVID-19 [19]. We established a statistical relationship between the disease severity and the presence of positive pulmonary symptoms such as dyspnoea and cyanosis (p<0.05). The most common extra-respiratory symptom in children is diarrhoea. In our study, this symptom was significantly more common (p < 0.05) in children with severe course of the disease: 42.10% versus 15.79% in children with moderate course. Tian et al. presented similar findings [20]. Available sources describe that among children, diarrhoea may be the first clinical manifestation of COVID-19, and faecal transmission of SARS-CoV-2 virus cannot be excluded [21].

Almost half of the children had at least one concomitant pathology. The most common were, chronic tonsillitis, in 11.8% of cases, and anaemia, in 6.5% of cases. Previous studies indicate that the presence of comorbidity may have a negative impact on the COVID-19 progression [22, 23]. Of the 37 severely ill children in our study, 19 had comorbidities, but we did not establish a statistical relationship between COVID-19 severity and the presence of comorbidities. In our study, children with recurrent respiratory infections (RRI) significantly more often progressed to a severe course rather than a moderate course. Further studies of the course of COVID-19 in children with RRIs are necessary.

Open sources describe a multitude of diverse blood test value changes in children with confirmed COVID-19, but generally without classification by age group, sex, or disease severity. Some preliminary studies identified leukopenia and leukocytosis in 7.3% and 10.7% of patients, respectively, as blood test variations in children with COVID-19 [15, 16, 24]. According to other reviews, the most common abnormalities in paediatric patients were leukopenia/lymphopenia and increased creatinine and lymphopenia/lymphocytosis, in 21% and 5% of cases, respectively [13]. In all likelihood, COVID-19 causes cytopenias, like other viral diseases [25]. In our study, leukopenia was reported in 22.22% of cases and lymphopenia in 68.42%, but we did not establish a statistical relationship between leukopenia/lymphopenia and severity of the disease (p> 0.05), although available sources claim that those indicators are associated with a worse prognosis for COVID-19 progression [26, 27]. We established a positive correlation (r=0.7 p<0.05) between the level of CRP and COVID-19 severity. Other researchers reported similar findings [28]. Thus, CRP can be considered one of the possible markers of the severe course of COVID-19. We established a correlation between the level of AST and COVID-19 severity (p<0.05). The same results can be found in other available sources [29]. This indicates that, when dealing with COVID-19, clinicians should closely monitor and evaluate vital organ function (kidney, liver, heart). The level of erythrocytes requires special attention. It is important to note that reduced RBC count is statistically more significant among children with severe COVID-19 compared to those with the moderate disease progression (p<0.05). The same findings were reported by Taneri et al. [30]. Furthermore, none of the children with elevated hemoglobin had severe progression of the disease, which may both confirm the importance of red blood in predicting the disease progression and point out to the red blood protective properties (p<0.05). No statistical difference could be established for other laboratory test values.

All children in our study underwent a chest X-ray because it is fast and easily available. At present, lung radiography is becoming the primary imaging modality for paediatric patients with moderate to severe COVID-19 progression, whereas CT scan is prescribed by doctor in case of progressive clinical deterioration [31, 32]. Changes in the lungs revealed through X-ray were observed in 76.61% of the examined children, yet 23.39% did not show any changes. In our study, as well as in other studies, ground-glass opacity symptom was recorded in 50.88% of patients. This symptom is characteristic of the SARS-CoV-2 virus [33]. A statistical difference was established between the disease severity and changes in the lungs (p=0.001).

It should be noted that our study is retrospective, based on a small cohort of patients, yet it may become a useful foundation for further research of COVID-19 progression in children in Ukraine.

## Conclusion

Our study showed that among hospitalized children in Ukraine, COVID-19 usually has a moderate progression and a good prognosis. However, a small share of children is likely to progress to severe disease that requires hospitalization in the intensive care unit. Gender does not play an important role in determining risk factors for COVID-19 severity among hospitalized children. SARS-CoV-2 was more common in school-age children. The family cluster has proven to be the most important epidemiological factor in the virus transmission. The clinical manifestations of COVID-19 among hospitalized children in Ukraine are similar to those caused by other viruses, with the most common symptoms being fever, dry cough, and sore throat. No specific symptoms peculiar to COVID-19 were identified. Ageusia and anosmia, typical of adult patients, are less common in children, yet they may be a marker of a favorable prognosis. Diarrhoea is one of the most common extra-pulmonary symptoms in children with COVID-19. Positive pulmonary symptoms, such as dyspnoea and cyanosis, are common in severely ill children. CRP can be considered as a likely marker of COVID-19 severity. In most cases, the SARS-CoV-2 virus causes typical ground-glass opacity seen on chest X-ray imaging of the lungs.

Globally, the emergence of the SARS-CoV-2 virus represents a new challenge in the field of healthcare. Data on the COVID-19 progression are being constantly updated, but further studies are necessary for better understanding of the course of the disease in pediatric patients.

### Keypoints

- Children in Ukraine more often had a moderate course of COVID-19.
- Schoolchildren of ages 6 to 18 were the predominant group across the age groups, representing 67.25% of all examined patients.
- The most common symptoms were fever in 88.2% of cases; sore throat in 69.2%; cough in 60.9%.
- Anosmia and ageusia, often observed in the adult population, are much less common in pediatric patients.
- Almost half of the children had at least one concomitant pathology. In particular, chronic tonsillitis was reported more frequently, in 11.8% of cases, as well as anaemia, in 6.5% of cases.
